# Single-Nucleotide Polymorphisms in the 3' Untranslated Region of *CORIN* Associated With Cardiovascular Diseases in a Chinese Han Population: A Case–Control Study

**DOI:** 10.3389/fcvm.2021.625072

**Published:** 2021-08-02

**Authors:** Yichang Zhao, Xiaoyang Yuan, Yang Zhong, Yutao Zhang, Shushan Zhang, Sisi Li, Yuanyuan Zhao, Wenjun Zheng, Jinqiu Liu, Yunlong Xia, Yanzong Yang, Ying Liu, Feifei Chen

**Affiliations:** ^1^Department of Cardiology, The First Affiliated Hospital of Dalian Medical University, Dalian, China; ^2^Department of Clinical Laboratory, The First Affiliated Hospital of Dalian Medical University, Dalian, China; ^3^Department of Cardiology, The Fifth People's Hospital of Dalian, Dalian, China; ^4^Chinese Center for Disease Control and Prevention, National Institute of Occupational Health and Poison Control, Beijing, China; ^5^Department of Ultrasonography, The Fifth Affiliated Hospital of Sun Yat-sen University, Zhuhai, China; ^6^Key Laboratory of Prevention and Treatment of Cardiovascular and Cerebrovascular Diseases of Ministry of Education, Department of Epidemiology, School of Public Health and Health Management, Gannan Medical University, Ganzhou, China; ^7^Key Laboratory of Organ Transplantation, National Health Commission (NHC) Key Laboratory of Organ Transplantation, Key Laboratory of Organ Transplantation, Ministry of Education, Chinese Academy of Medical Sciences, Institute of Organ Transplantation, Tongji Medical College, Tongji Hospital, Huazhong University of Science and Technology, Wuhan, China; ^8^Department of Cardiovascular Surgery, The First Affiliated Hospital of Dalian Medical University, Dalian, China

**Keywords:** *CORIN*, single nucleotide polymorphisms, lipid levels, cardiovascular diseases, hsa-miR-494-3p

## Abstract

**Background:** Corin is a transmembrane serine protease that activates pro-forms of atrial and brain natriuretic peptides. Numerous studies have indicated that corin played an important role in cardiovascular diseases (CVDs). However, there have been few studies about the correlation between single-nucleotide polymorphisms (SNPs) in the 3' untranslated region (3'UTR) of *CORIN* and CVDs. The aims of this study were to investigate the associations of three SNPs (rs3749585, rs4695253, and rs12641823) in the 3'UTR of *CORIN* with CVDs and to find the seed regions of microRNAs (miRNAs) that bind to SNPs of *CORIN*.

**Methods and Results:** A case–control study (*n* = 3,537) was performed in a Han population of northeastern China. CVDs included essential hypertension (EH), atrial fibrillation (AF), heart failure (HF), and coronary artery disease (CAD). Genotyping was performed using high-resolution melt analysis. In the EH-control study, rs3749585^T^ was significantly associated with the risk of EH after adjusting for sex and age in allelic (*p*_*adj*_ = 0.049; *OR*: 1.113) and dominant (*p*_*adj*_ = 0.015, *OR*: 1.233) models. Rs4695253^T^ was significantly associated with the risk of EH in the recessive model after adjusting for sex and age (*p*_*adj*_ = 0.005, *OR*: 2.084). Rs3749585^T^ was significantly and negatively associated with AF in the dominant and additive models after adjusting for sex, age, EH, HF, T2DM, and CAD (dominant: *p*_*adj*_ = 0.009, *OR*: 0.762; additive: *p*_*adj*_ = 0.048, *OR*: 0.873). In the HF-control study and CAD-control study, none of the three SNPs was associated with HF and CAD after adjusting for covariates in any models (*p*_*adj*_ > 0.05). The levels of high-density lipoprotein (HDL) in rs4695253^CC+CT^ were lower than the levels of HDL in rs4695253^TT^ (42.47 ± 10.30 vs. 48.0 ± 10.24 mg/dl, *p*_*adj*_ = 0.008). The levels of total cholesterol (TC) in rs4695253^CC+CT^ were lower than the levels of TC in rs4695253^TT^ (164.01 ± 49.15 vs. 180.81 ± 43.92 mg/dl, *p*_*adj*_ = 0.036). Luciferase assay revealed that the relative luciferase activity of rs3749585^CC^-transfected cells was significantly decreased by miR-494-3p, in comparison to cells transfected with rs3749585^TT^ (*p* < 0.001). A significant decrease in the relative luciferase activity of rs3749585^TT^ reporter was observed as compared with rs3749585^CC^ reporter in the presence of miR-1323 or miR-548o-3p (*p* = 0.017 and 0.012, respectively).

**Conclusions:** We found significant associations between rs3749585^T^ and rs4695253^T^ and EH, between rs4695253^T^ and the levels of TC and HDL, and between rs3749585^T^ and AF. Hsa-miR-494-3p may serve as a potential therapeutic target for EH and AF patients in the future.

## Introduction

Corin is a transmembrane serine protease made primarily in atrial and ventricular cardiomyocytes (1, 2) and converts pro-atrial natriuretic peptide (pro-ANP) and pro-brain natriuretic peptide (pro-BNP) into biologically active ANP and BNP in cell-based studies (3), the hormones that regulate blood pressure by promoting natriuresis, diuresis, and vasodilatation (4, 5) and suppress renin and endothelin release (6). ANP is also known for its ability to modulate the electrical activities of the heart, its effects on specific ion channels (7, 8), and its suppressive effect on cardiac fibroblast growth during cardiac hypertrophy (9). In humans, *CORIN* variants (I555/P568) are associated with hypertension and cardiac hypertrophy (10–12). Heart failure (HF) patients with the two variants had impaired pro-BNP processing and poor clinical outcomes (13). In mice, lack of corin prevented natriuretic peptide processing and resulted in hypertension (14); corin deficiency caused cardiac hypertrophy during pregnancy (15). Moreover, in many clinical studies, serum or plasma soluble corin concentrations were changed in the patients with cardiovascular diseases (CVDs) and the levels of soluble corin were associated with the risk and prognosis of CVDs. Plasma concentrations of corin were decreased in HF (16, 17) and hypertensive patients (18). Patients with atrial fibrillation (AF) were found to have higher serum corin levels than those without AF (19). A large study involving 856 patients with acute myocardial infarction and 856 healthy subjects reported that serum corin concentrations were significantly decreased in the former (20). Plasma corin concentrations, assessed 2 days after reperfused ST-segment elevation myocardial infarction, correlated with infarct size as assessed by cardiac magnetic resonance (21). Furthermore, plasma corin can be a biomarker for the prognosis of acute myocardial infarction (22) and HF (23). The significance of corin seems more important in HF. HF is associated not only with reduced plasma corin levels (16, 17, 24) and decreased myocardial *CORIN* expression (25), but also with impaired cleavage of pro-ANP (17). In mice with progressive dilated or ischemic cardiomyopathy, restoration of suppressed cardiac corin expression levels reduces myocardial fibrosis, improves contractile function, reduces alveolar edema and congestion, and attenuates heart failure (26–29).

Previous studies have demonstrated the association between *CORIN* variants and CVDs. Most of them mainly focused on the exons and the intron/exon boundaries (30–50 nucleotides into the intronic regions) (30, 31). However, the association between single-nucleotide polymorphisms (SNPs) in the 3' untranslated regions (3' UTR) of *CORIN* and CVDs is unknown. Two studies reported the correlation between a SNP, rs3749585, which is located in the 3'UTR of *CORIN*, and hypertension, but the results were not conclusive (32, 33).

Posttranscriptional regulation of gene expression by microRNAs (miRNAs) requires base-pairing between miRNAs and target mRNAs. Target recognition is crucially dependent on Watson–Crick base-pairing between the seed region of miRNA and miRNA recognition elements located in the 3' UTR of target mRNAs. The resulting translational inhibition or mRNA degradation contributes to the downregulation of the corresponding target protein (34, 35). Cardiac-specific miRNAs have been shown to regulate a number of critical cellular functions in the developing and normal adult heart and CVDs. If there are allelic variants in the miRNA recognition elements of *CORIN* 3' UTR, different kinetics of *CORIN* downregulation may exist. The aims of this study are to evaluate the association between SNPs in the 3' UTR of *CORIN* and CVDs and to find the seed regions of miRNAs that bind to SNPs of *CORIN*.

## Materials and Methods

### Study Subjects

The study subjects are from the Gene ID population, which is a large current Chinese database with clinical data and blood samples from >10,000 Chinese northeastern patients and controls that can be used to identify susceptibility genes for various CVDs. Written informed consent was obtained from the participants when they agreed to be enrolled in the Gene ID database. All of the subjects were of Han ethnic origin by self-description. This study was approved by the Ethics Committee of The First Affiliated Hospital of Dalian Medical University and conformed to the guidelines set forth by the Declaration of Helsinki.

CVDs in the study included essential hypertension (EH), AF, HF, coronary artery disease (CAD), and left ventricular hypertrophy (LVH). The diagnostic criteria for EH were in accordance with 2018 Chinese Guidelines for Prevention and Treatment of Hypertension (36) and defined as a systolic blood pressure of ≥140 mmHg or a diastolic blood pressure of ≥90 mmHg. The diagnostic criteria for AF were in accordance with 2019 AHA/ACC/HRS Focused Update of the 2014 AHA/ACC/HRS guideline for the Management of Patients With Atrial Fibrillation (37) and diagnosis determined using ECG or Holter recordings, regardless of clinical symptoms. The diagnostic criteria for HF were in accordance with Chinese guidelines for the diagnosis and treatment of heart failure 2018 (38) and the etiologies of HF in the study included EH, AF, and CAD. The diagnostic criteria for CAD were from 2015 ESC guidelines for the management of acute coronary syndromes in patients and 2019 ESC guidelines for the diagnosis and management of chronic coronary syndromes (39, 40).

LVH was diagnosed if the thickness of the left ventricular posterior wall (LVPW) and interventricular septum (IVS) was >12 mm and the ratio of IVS to LVPW was <1.3. Any patients with secondary hypertension, thyroid disorder, valvular heart disease (causing secondary AF), aortic valve stenosis and hypertrophic cardiomyopathy (causing ventricular hypertrophy), or HF etiologies other than EH, AF, and CAD were excluded. Each subject was evaluated by symptoms, blood pressure, BNP, electrocardiogram, echocardiogram, and computed tomography coronary angiography, and the subjects in the control group were free from the abovementioned CVDs.

The clinical data included age, gender, type 2 diabetes mellitus (T2DM), total cholesterol (TC), high-density lipoprotein (HDL), low-density lipoprotein (LDL), and triglyceride (TG) in both groups.

### SNP Selection, Genotyping, and miRNA Prediction

There are 27 SNPs in the 3'UTR of *CORIN* according to Ensembl database (http://asia.ensembl.org/index.html). Excluding the SNPs with minor allele frequency (MAF) <5% (http://www.ncbi.nlm.nih.gov/snp/), we finally selected four SNPs, namely, rs3749585, rs3749584, rs4695253, and rs12641823. MiRNASNP v3 (http://bioinfo.life.hust.edu.cn/miRNASNP/) was used to search for miRNAs of which the seed regions bind to SNPs and to explore the impact of the SNPs on mRNA–miRNA interaction.

Genomic DNA was isolated from peripheral blood lymphocytes with TIANamp Blood DNA Kit (TiangenBiotect, Beijing, China) according to the manufacturer's protocols. Polymerase chain reaction (PCR) was performed in a 20-μl final volume containing 10 μl of high-resolution melting (HRM) master mix (Roche Diagnostics, Basel, Switzerland), 0.5 μM each primer (Supplementary Table 1), 2.5 mM Mg^2+^, and 25 ng of human genomic DNA template. PCR and HRM were performed on a LightCycler® 480 II (Roche, Basel, Switzerland) with the following thermal profiles: PCR was 95°C for 10 s; 45 cycles of 95°C for 10 s, primer-specific annealing temperature (Supplementary Table 1) for 15 s, and 72°C for 15 s. Then, HRM cycle was 95°C for 1 min, 40°C for 1 min, 65°C for 1 s, and 40°C for 10 s. During each run of reaction, three positive control DNA samples with known genotypes (major homozygous, heterozygous, and minor homozygous), as well as a negative control with DNA template replaced by ddH_2_O were included. To validate the accuracy of HRM genotyping data, 10 samples for each genotype of SNPs were randomly selected for direct sequencing (Sangon Biotech, Shanghai, China). All sequencing results were consistent with the genotypes as determined by HRM analysis.

### Cell Culture, Plasmids, and Transfection

HEK293 cells were purchased from the American Type Culture Collection. Cells were cultured in Dulbecco's modified Eagle's medium supplemented with 10% fetal bovine serum in a humidified incubator with 5% CO_2_ at 37°C.

The sequence of human *CORIN* encompassing rs3749585(T/C) was GACTGTGAAGAGCTGCCTGCAGAGAGCTGTACAGAAGCACTTTTCATGGACAGAAATGCTCAATCGTGCACTGCAAATTTGCATGTTTGTTT
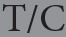
GGACTAATTTTTTTCAATTTATTTTTTCACCTTCATTTTTCTCTTATTTCAAGTTCAATGAAAGACTTTACAAAAGCAAACAAAGCAGACT. The sequences of rs3749585 TT and CC were synthesized and were inserted into PUC57 plasmid by GenScript Company (Nanjing, China), referred to as PUC57-*CORIN*-rs3749585-TT and PUC57-*CORIN*-rs3749585-CC, respectively. The PUC57-*CORIN*-rs3749585-TT and PUC57-*CORIN*-rs3749585-CC were digested with *Xho*I and *Not*I, and the PCR products were subcloned into the pYr-MirTarget luciferase plasmid, resulting in pYr-MirTarget-*CORI*N-rs3749585-TT and pYr-MirTarget-*CORI*N-rs3749585-CC. The primers used for Colony PCR were 5'-GGTTCTTTTCCAACGCTATT-3' (forward) and 5'-GACTCATTTAGATCCTCACAC-3' (reverse).

MiRNAs mimics were used to upregulate the expression of miRNAs, and negative control (NC) was used as control. MiRNAs were purchased from GenePharma Limited Company (Shanghai, China).

### Dual-Luciferase Reporter Assay

HEK293 cells (2 × 10^5^ cells/well) were maintained in 24-well plates and co-transfected with luciferase reporter plasmids and mimics using Lipofectamine 2000 Transfection reagent (Thermo Fisher Scientific, Waltham, MA, USA). After 24 h of incubation at 37°C with 5% CO_2_, the firefly luciferase activity was measured through the Dual-Glo luciferase report system (Beyotime Biotechnology, Shanghai, China) and calculated by normalization to the Renilla luciferase control.

### Statistical Analysis

Statistical power analysis of study populations was conducted with the program PS (Power and Sample size Calculations, version 3.0.43). Hardy–Weinberg equilibrium (HWE) was tested in the control group with PLINK, version 1.07 (41) and the significance level was set at 0.001. Pearson's chi-squared (χ^2^) and unpaired Student's *t*-tests were performed with SPSS version 22.0 software (IBM Incorporation, Armonk, New York, USA) for categorical traits (gender, EH, AF, HF, CAD, LVH, and T2DM) and continuous traits (age, TC, TG, LDL, and HDL), respectively. For allelic association analysis, 2 × 2 contingency tables assessed by Pearson's chi-squared (χ^2^) test were used to compare differences in the MAF of the SNPs between the case and control groups (PLINK v1.07). Odds ratios (*ORs*) and corresponding 95% confidential intervals (95% CI) were also calculated. Genotypic association analyses under three genetic models (dominant, recessive, and additive) were performed using 2 × 3 contingency tables assessed by Pearson's chi-squared (χ^2^) test (PLINK v1.07). Multiple logistic regression analysis was used to adjust for covariates and the covariates adjusted for were different for each CVD. The EH group was corrected for sex and age; the AF group was corrected for sex, age, EH, HF, T2DM, and CAD; the HF group was corrected for sex, age, EH, CAD, AF, and T2DM; the CAD group was corrected for sex, age, EH, and T2DM. When the hypertensive subjects were divided into the LVH subgroup and the non-LVH subgroup, basic statistical methods were applied as described. Associations between SNPs and lipid levels under allelic and genetic models were analyzed by linear logistic regression; β and 95% CI were also calculated. In addition, we performed one-way ANOVA analysis to test the ratios of dual-luciferase activity in different groups. Two-tailed *p* < 0.05 was accepted as statistically significant.

## Results

### Clinical Characteristics

There was no deviation from the HWE for rs3749585, rs4695253, and rs12641823 in either case or control groups (*p* > 0.001, Supplementary Table 2). Because rs3749584 deviated significantly from the HWE, we did not show the associations between rs3749584 and CVDs. The total included 3,537 subjects. In the EH group, there were 1,495 cases and 2,042 controls; in the AF group, there were 618 cases and 2,919 controls; in the HF group, there were 464 cases and 3,073 controls; in the CAD group, there were 715 cases and 2,822 controls. The mean age of the patients in each group was older than that of the controls (*p* < 0.001, Table 1). The proportion of T2DM in the case group was higher than that in the control group (*p* < 0.001, Table 1). The distribution of gender between cases and controls in each group was similar (*p* > 0.05, Table 1).

**Table 1:** Clinical characteristics of study population.

**Characteristics**	**EH**			**AF**			**HF**			**CAD**		
	**Case**	**Control**	***P***	**Case**	**Control**	***P***	**Case**	**Control**	***P***	**Case**	**Control**	***P***
Total number of samples	1,495	2,042		618	2,919		464	3,073		715	2,822	
Age, years (M ± SD)	66.39 ± 11.58	52.78 ± 16.64	**<0.001**	68.59 ± 11.44	56.41 ± 16.23	**<0.001**	70.59 ± 10.94	56.71 ± 16.06	**<0.001**	68.00 ± 10.63	56.14 ± 16.46	**<0.001**
Sex (male/female)	778/717	1,020/1,022	0.22	336/282	1,463/1,456	0.057	236/228	1,563/1,510	0.995	435/280	1,364/1,458	**<0.001**
TC (mg/dl, M ± SD)	156.47 ± 50.43	175.16 ± 40.55	**<0.001**	162.46 ± 48.92	167.4 ± 43.95	0.029	156.69 ± 48.56	165.59 ± 47.28	0.001	149.91 ± 50.92	179.17 ± 44.6	**<0.001**
HDL (mg/dl, M ± SD)	42.5 ± 10.65	43.32 ± 12.77	0.115	40.96 ± 14.08	43.51 ± 10.36	**<0.001**	40.58 ± 15.16	43.41 ± 10.31	**<0.001**	41.54 ± 14.09	43.43 ± 10.06	0.002
LDL (mg/dl, M ± SD)	122.74 ± 81.57	101.77 ± 30.02	**<0.001**	100.64 ± 40.7	119.63 ± 73.87	**<0.001**	111.25 ± 66.72	115.37 ± 67.24	0.26	128.43 ± 93.65	108.13 ± 49.08	**<0.001**
TG (mg/dl, M ± SD)	144.66 ± 106.49	125.65 ± 89.81	**<0.001**	117.58 ± 75.93	144.4 ± 107.5	**<0.001**	119.19 ± 76.8	141.55 ± 104.787	**<0.001**	151.36 ± 120.95	130.68 ± 89.18	**<0.001**
T2DM (0/1)	993/502	1,884/158	**<0.001**	454/164	2,423/496	**<0.001**	304/160	2,573/500	**<0.001**	449/266	2,428/394	**<0.001**
EH (0/1)	N/A	N/A	N/A	216/402	1,826/1,093	**<0.001**	100/364	1,942/1,131	**<0.001**	180/535	1,862/960	**<0.001**
AF (0/1)	1,093/402	1,826/216	**<0.001**	N/A	N/A	N/A	166/298	2,753/320	**<0.001**	559/156	2,360/462	**0.001**
HF (0/1)	1,131/364	1,942/100	**<0.001**	320/298	2,753/166	**<0.001**	N/A	N/A	N/A	539/176	2,534/288	**<0.001**
CAD(0/1)	960/535	1,862/180	**<0.001**	462/156	2,360/559	**0.001**	288/176	2,534/539	**<0.001**	N/A	N/A	N/A

*EH, essential hypertension; AF, atrial fibrillation; HF, heart failure; CAD, coronay artery disease; T2DM, type 2 diabetes mellitus; TC, total cholesterol; HDL, high density lipoprotein; LDL, low density lipoprotein; TG, triglyceride; N/A, not applicable; M ± SD, means ± standard deviation. The bold values mean P < 0.05*.

### Analysis of Associations of SNPs in the 3' UTR of *CORIN* With EH

In the EH-control study, only rs3749585^T^ was significantly associated with the risk of EH [*p*_*adj*_ = 0.049; *OR*: 1.113 (95% CI, 1.00–1.238); Table 2] after adjusting for covariates of sex and age in the allelic model. SNP rs3749585^T^ was significantly associated with the risk of EH in the dominant model after adjusting for covariates sex and age [*p*_*adj*_ = 0.015, *OR*: 1.233 (95% CI, 1.042–1.459); Table 3]. SNP rs4695253^T^ was significantly associated with the risk of EH in the recessive model after adjusting for covariates sex and age [*p*_*adj*_ = 0.005, *OR*: 2.084 (95% CI, 1.246–3.488); Table 3]. SNP rs12641823^A^ failed to show any significant association with risk of EH in the study (*p*_*adj*_ > 0.05, Tables 2, 3) under allelic and genotypic inheritance models.

**Table 2:** Analysis of allelic association of SNPs in 3' UTR of *CORIN* with essential hypertension.

**SNP**	**Sample size (total *n* = 3,537)**	**Risk allele**	**Frequency**	**Without adjustment**		**With adjustment**	
	**Case/control**		**(case/control)**	***P_***obs***_***	**OR (95% CI)**	***P_***adj***_***	**OR (95% CI)**
rs3749585	1,495/2,042	T	0.483/0.454	**0.015**	**1.127 (1.023–1.241)**	**0.049**	**1.113 (1.000–1.238)**
rs4695253		T	0.152/0.132	**0.020**	**1.118 (1.027–1.356)**	0.052	1.164 (0.999–1.357)
rs12641823		A	0.457/0.462	0.703	0.981 (0.887–1.084)	0.988	0.999 (0.894–1.116)

*SNP, single-nucleotide polymorphism; 3'UTR, 3 untranslated region; OR, odds ratio; CI, confidential interval; P_adj_, P-value for association after adjusting for covariates sex and age by multiple logistic regression analysis using SPSS version 22.0; P_obs_, P-value for association before adjusting for covariates age and sex by 2 × 2 contingence tables using PLINK version 1.07. The bold values mean P < 0.05*.

**Table 3:** Analysis of genotypic association of SNPs in 3' UTR of *CORIN* with essential hypertension under different genetic inheritance models.

**SNP**	**Model**	**Without adjustment**		**With adjustment**	
		***P_***obs***_***	**OR (95% CI)**	***P_***adj***_***	**OR (95% CI)**
rs3749585	Dominant	3.30E-04	1.322 (1.135–1.540)	**0.015**	**1.233 (1.042–1.459)**
	Recessive	0.878	1.013 (0.860–1.193)	0.492	1.065 (0.889–1.276)
	Additive	0.017	1.123 (1.021–1.236)	0.050	1.112 (1.000–1.237)
rs4695253	Dominant	0.088	1.146 (0.980–1.340)	0.218	1.115 (0.938–1.325)
	Recessive	0.008	1.880 (1.181–2.994)	**0.005**	**2.084 (1.246–3.488)**
	Additive	0.021	1.176 (1.024–1.349)	0.054	1.161 (0.998–1.352)
rs12641823	Dominant	0.355	0.930 (0.798–1.805)	0.499	0.943 (0.796–1.118)
	Recessive	0.708	1.033 (0.871–1.225)	0.470	1.072 (0.88–1.293)
	Additive	0.707	0.981 (0.889–1.083)	0.989	0.999 (0.896–1.114)

*SNP, single-nucleotide polymorphism; 3'UTR, 3 untranslated region; OR, odds ratio; CI, confidential interval; P_adj_, P-value for association after adjusting for covariates sex and age by multiple logistic regression analysis using SPSS version 22.0; P_obs_, P-value for association before adjusting for covariates age and sex by 2 × 2 contingence tables using PLINK version 1.07. The bold values mean P < 0.05*.

We divided the hypertensive group into the LVH subgroup (*n* = 590) and the non-LVH subgroup (*n* = 905) according to echocardiography. We found that all the three SNPs, rs3749585^T^, rs4695253^T^, and rs12641823^A^, were unrelated to the risk of LVH in the hypertensive group after adjusting for sex and age both under allelic and genetic models (*p*_*adj*_ > 0.05, Supplementary Tables 3, 4).

### Analysis of Allelic and Genotypic Associations of SNPs in the 3' UTR of *CORIN* With AF, HF, and CAD

In the AF-control study, HF-control study, and CAD-control study, all the three SNPs, rs3749585^T^, rs4695253^T^, and rs12641823^A^, were unrelated to the risk of AF, HF, and CAD after adjusting for covariates (*p*_*adj*_ > 0.05, Supplementary Table 5).

SNP rs3749585^T^ was significantly and negatively associated with AF in the dominant and additive models after adjusting for covariates sex, age, EH, HF, T2DM, and CAD [dominant: *p*_*adj*_ = 0.009, *OR*: 0.762 (95% CI, 0.621–0.935); additive: *p*_*adj*_ = 0.048, *OR*: 0.873 (95% CI, 0.763–0.999); Table 4]. SNP rs4695253^T^ and rs12641823^A^ were unrelated to AF after adjusting for covariates (*p*_*adj*_ > 0.05, Table 4) in three inheritance models.

**Table 4:** Analysis of genotypic association of SNPs in 3' UTR of *CORIN* with AF, HF, and CAD under different genetic inheritance models.

**Disease**	**SNP**	**Model**	**Without adjustment**		**With adjustment**	
			***P_***obs***_***	**OR (95% CI)**	***P_***adj***_***	**OR (95% CI)**
AF	rs3749585	Dominant	0.196	0.881 (0.728–1.067)	**0.009**	**0.762 (0.621–0.935)**
		Recessive	0.422	0.916 (0.738–1.136)	0.584	0.938 (0.746–1.179)
		Additive	0.196	0.921 (0.814–1.043)	**0.048**	**0.873 (0.763–0.999)**
	rs4695253	Dominant	0.479	1.075 (0.879–1.315)	0.834	1.023 (0.826–1.267)
		Recessive	0.129	1.517 (0.885–2.600)	0.264	1.390 (0.780–2.474)
		Additive	0.281	1.101 (0.924–1.312)	0.599	1.051 (0.873–1.266)
	rs12641823	Dominant	0.391	0.917 (0.753–1.117)	0.585	0.943 (0.765–1.164)
		Recessive	0.192	0.860 (0.685–1.079)	0.240	0.866 (0.680–1.101)
		Additive	0.193	0.918 (0.808–1.044)	0.305	0.931 (0.813–1.067)
HF	rs3749585	Dominant	0.113	1.201 (0.957–1.506)	0.110	1.252 (0.951–1.648)
		Recessive	0.993	0.999 (0.786–1.269)	0.478	1.111 (0.831–1.486)
		Additive	0.315	1.074 (0.934–1.235)	0.150	1.135 (0.955–1.349)
	rs4695253	Dominant	0.019	1.304 (1.046–1.627)	0.068	1.286 (0.981–1.685)
		Recessive	0.402	1.307 (0.599–2.446)	0.934	0.968 (0.454–2.066)
		Additive	0.020	1.256 (1.036–1.521)	0.121	1.202 (0.853–1.518)
	rs12641823	Dominant	0.906	1.014 (0.810–1.268)	0.617	1.071 (0.819–1.399)
		Recessive	0.260	0.864 (0.669–1.115)	0.559	0.914 (0.675–1.236)
		Additive	0.565	0.959 (0.831–1.106)	0.989	0.999 (0.841–1.186)
CAD	rs3749585	Dominant	0.355	1.093 (0.905–1.319)	0.721	0.963 (0.781–1.186)
		Recessive	0.090	0.834 (0.676–1.029)	0.119	0.833 (0.662–1.048)
		Additive	0.688	0.976 (0.867–1.099)	0.250	0.925 (0.809–1.057)
	rs4695253	Dominant	0.336	0.907 (0.743–1.107)	0.116	0.839 (0.675–1.044)
		Recessive	0.702	0.888 (0.485–1.627)	0.293	0.707 (0.370–1.349)
		Additive	0.336	0.917 (0.768–1.094)	0.090	0.847 (0.699–1.026)
	rs12641823	Dominant	0.756	0.970 (0.803–1.173)	0.884	1.016 (0.824–1.252)
		Recessive	0.986	1.002 (0.812–1.237)	0.823	1.027 (0.814–1.295)
		Additive	0.850	0.988 (0.875–1.116)	0.824	1.015 (0.88–1.161)

*SNP, single-nucleotide polymorphisms; 3'UTR, 3 untranslated region; OR, odds ratio; CI, confidential interval; AF, atrial fibrillation; HF, heart failure; CAD, coronay artery disease; P_adj_, P-value for association after adjusting for covariates by multiple logistic regression analysis using SPSS version 22.0; P_obs_, P-value for association before adjusting for covariates by 2 × 2 contingence tables using PLINK version 1.07. The bold values mean P < 0.05*.

In the HF-control study and CAD-control study, none of the three SNPs, rs3749585^T^, rs4695253^T^, and rs12641823^A^, was associated with HF or CAD after adjusting for covariates in three inheritance models (*p*_*adj*_ > 0.05, Table 4).

### Analysis of Associations of SNPs in the 3' UTR of *CORIN* With Lipid Levels

Lipid levels included TC, TG, LDL, and HDL. We found no association between any of the three SNPs, rs3749585^T^, rs4695253^T^, and rs12641823^A^, and lipid levels in the allelic model (*p*_*adj*_ > 0.05, Supplementary Table 6).

**Figure 1:**
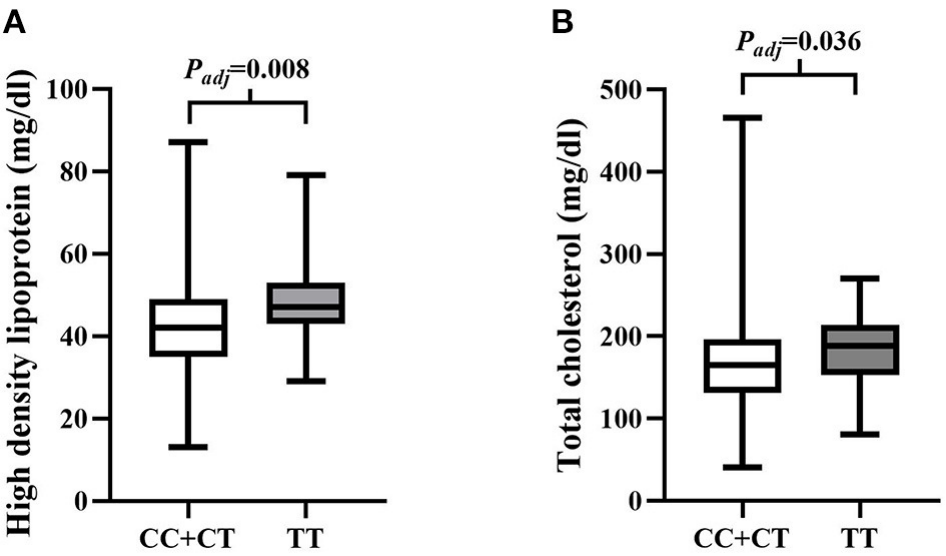
Analysis of association of rs4595253 with the levels of high density lipoprotein (HDL) and total cholesterol (TC). **(A)** The levels of HDL for rs4695253^CC+CT^ were lower than the levels of HDL for rs4695253^TT^ (42.47 ± 10.30 vs. 48.0 ± 10.24 mg/dl, *P*_adj_ = 0.008). **(B)** The levels of TC for rs4695253^CC+CT^ were lower than the levels of TC for rs4695253^TT^ (164.01 ± 49.15 vs. 180.81 ± 43.92 mg/dl, *P*_adj_ = 0.036).

SNP rs4695253^T^ was significantly associated with the levels of HDL and TC in the recessive model after adjusting for covariates sex and age [HDL: *p*_*adj*_ = 0.008, β: 5.053 (95% CI, 1.331–8.775); TC: *p*_*adj*_ = 0.036, β: 15.944 (95% CI, 1.050–30.837); Table 5]. The levels of HDL for rs4695253^CC+CT^ were lower than the levels of HDL for rs4695253^TT^ (42.47 ± 10.30 vs. 48.0 ± 10.24 mg/dl, *p*_*adj*_ = 0.008, Figure 1A). In addition, the levels of TC for rs4695253^CC+CT^ were lower than the levels of TC for rs4695253^TT^ (164.01 ± 49.15 vs. 180.81 ± 43.92 mg/dl, *p*_*adj*_ = 0.036, Figure 1B). SNP rs3749585^T^ and rs12641823^A^ failed to show any significant association with lipid levels in any genetic inheritance models (*p*_*adj*_ > 0.05, Table 5).

**Table 5:** Analysis of genotypic association of SNPs in 3' UTR of *CORIN* with lipid levels under different genetic inheritance models.

**Lipid**	**SNP**	**Model**	**Without adjustment**		**With adjustment**	
			***P_***obs***_***	**β (95% CI)**	***P_***adj***_***	**β (95% CI)**
TG	rs3749585	Dominant	0.117	(−8.849) (−19.921 to 2.224)	0.142	(−8.266) (−19.293 to 2.762)
		Recessive	0.206	(−7.706) (−19.664 to 4.253)	0.166	(−8.406) (−20.307 to 3.496)
		Additive	0.080	(−6.317) (−13.395 to 0.760)	0.079	(−6.318) (−13.361 to 0.724)
	rs4695253	Dominant	0.426	4.079 (−5.960 to 14.118)	0.374	4.529 (−5.467 to 14.525)
		Recessive	0.305	15.683 (−14.305 to 45.671)	0.263	17.037 (−12.812 to 46.885)
		Additive	0.314	4.552 (−4.313 to 13.417)	0.264	5.027 (−3.804 to 13.857)
	rs12641823	Dominant	0.716	1.769 (−7.754 to 11.291)	0.228	1.101 (−8.378 to 10.580)
		Recessive	0.935	0.475 (−10.947 to 11.897)	0.958	(−0.303) (−11.666 to 11.060)
		Additive	0.776	0.910 (−5.359 to 7.178)	0.904	0.385 (−5.852 to 6.622)
HDL	rs3749585	Dominant	0.395	(−0.515) (−1.702 to 0.671)	0.273	(−0.655) (−1.825 to 0.515)
		Recessive	0.171	(−0.896) (−2.178 to 0.387)	0.173	(−0.879) (−2.144 to 0.385)
		Additive	0.176	(−0.524) (−1.283 to 0.235)	0.132	(−0.575) (−1.323 to 0.173)
	rs4695253	Dominant	0.156	0.910 (−0.349 to 2.169)	0.255	0.636 (−0.523 to 1.973)
		Recessive	0.004	5.500 (1.745 to 9.255)	**0.008**	**5.053 (1.331–8.775)**
		Additive	0.036	1.190 (0.079 to 2.301)	0.073	1.008 (−0.094 to 2.110)
	rs12641823	Dominant	0.703	(−0.228) (−1.401 to 0.945)	0.504	(−0.396) (−1.557 to 0.766)
		Recessive	0.637	0.339 (−1.069 to 1.747)	0.576	0.397 (−0.996 to 1.790)
		Additive	0.993	0.003 (−0.769 to 0.776)	0.894	(−0.052) (−0.817 to 0.713)
TC	rs3749585	Dominant	0.467	(−1.944) (−7.187 to 3.299)	0.356	(−2.439) (−7.615 to 2.737)
		Recessive	0.414	(−2.364) (−8.033 to 3.305)	0.403	(−2.386) (−7.980 to 3.208)
		Additive	0.343	(−1.623) (−4.976 to 1.730)	0.278	(−1.831) (−5.138 to 1.477)
	rs4695253	Dominant	0.227	3.110 (−1.937 to 8.156)	0.344	2.411 (−2.580 to 7.402)
		Recessive	0.022	17.597 (2.533 to 32.660)	**0.036**	**15.944 (1.050–30.837)**
		Additive	0.081	3.963 (−0.492 to 8.418)	0.145	3.277 (−1.131 to 7.686)
	rs12641823	Dominant	0.730	0.829 (−3.886 to 5.545)	0.988	0.034 (−4.623 to 4.692)
		Recessive	0.909	(−0.331) (−5.991 to 5.328)	0.946	(−0.193) (−5.778 to 5.393)
		Additive	0.870	0.260 (−2.845 to 3.365)	0.978	(−0.043) (−3.108 to 3.022)
LDL	rs3749585	Dominant	0.745	(−18.221) (−128.233 to 91.790)	0.710	(−20.833) (−130.874 to 82.209)
		Recessive	0.736	(−20.462) (−139.349 to 98.424)	0.776	(−17.250) (−136.085 to 101.585)
		Additive	0.684	(−14.615) (−84.959 to 55.730)	0.685	(−14.541) (−84.845 to 55.763)
	rs4695253	Dominant	0.725	(−17.549) (−115.543 to 80.444)	0.696	(−19.569) (−117.665 to 78.527)
		Recessive	0.684	(−60.645) (−353.296 to 232.005)	0.656	(−66.509) (−359.358 to 226.340)
		Additive	0.667	(−18.991) (−105.531 to 67.550)	0.633	(−21.100) (−107.767 to 65.567)
	rs12641823	Dominant	0.763	(−14.567) (−109.422 to 80.288)	0.816	(−11.253) (−106.165 to 83.659)
		Recessive	0.882	(−8.624) (−122.361 to 105.113)	0.920	(−5.805) (−119.537 to 107.926)
		Additive	0.780	(−8.914) (−71.363 to 53.535)	0.835	(−6.624) (−69.081 to 55.834)

*SNP, single-nucleotide polymorphisms; 3'UTR, 3 untranslated region; CI, confidential interval; TG, triglyceride; HDL, high density lipoprotein; TC, total cholesterol; LDL, low density lipoprotein; P_adj_, P-value for association after adjusting for covariates by multiple logistic regression analysis using SPSS version 22.0; P_obs_, P-value for association before adjusting for covariates by linear logistic regression using PLINK version 1.07. The bold values mean P < 0.05*.

### The Seed Regions of miRNAs Binding to SNPs in the 3'UTR of *CORIN* by Bioinformatic Analysis

**Figure 2:**
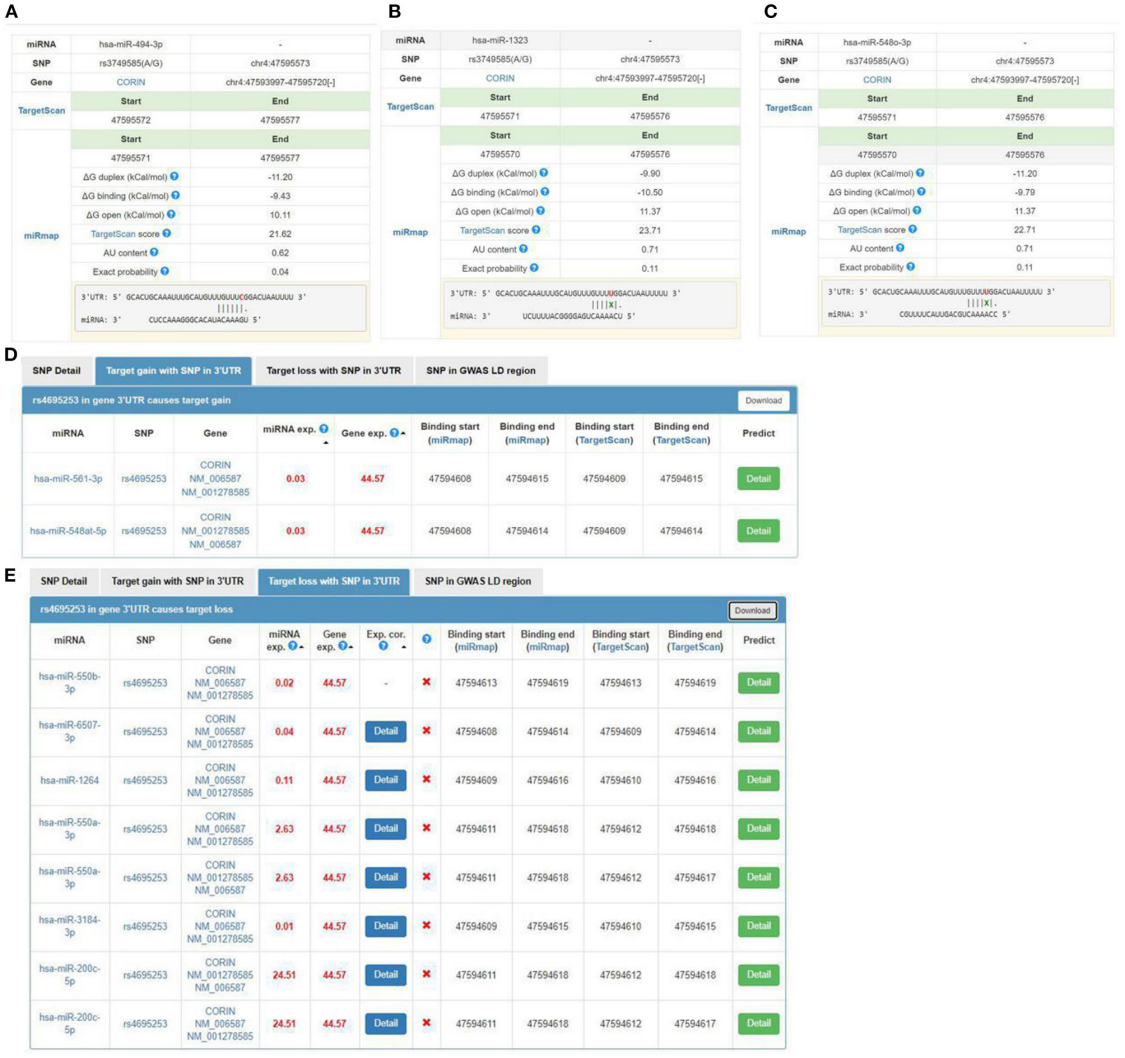
The seed regions of miRNAs that bind to rs3749585 and rs4695253 of *CORIN*. **(A)** hsa-miR-494-3p gains target with rs3749585; **(B,C)** hsa-miR-1323 and hsa-548o-3p lose target with rs3749585; **(D)** hsa-miR-561-3p and hsa-miR-548at-5p gain target with rs4695253; **(E)** hsa-miR-550b-3p, hsa-miR-6507-3p, hsa-miR-1264, hsa-miR-550a-3p, hsa-miR-3184-3p and hsa-miR-200c-5p lose target with rs4695253.

We used miRNASNP v3 to examine target gain and loss by rs3749585, rs4695253, and rs12641823 in miRNAs seed regions. Target gain with rs3749585 was predicted for hsa-miR-494-3p (Figure 2A); target loss with rs3749585 was predicted for hsa-miR-1323 and hsa-miR-548o-3p (Figures 2B,C). Target gain with rs4695253 was expected for hsa-miR-561-3p and hsa-miR-548at-5p (Figure 2D); target loss with rs4695253 was expected for hsa-miR-550b-3p, hsa-miR-6507-3p, hsa-miR-1264, hsa-miR-550a-3p, hsa-miR-3184-3p, and hsa-miR-200c-5p (Figure 2E). Target gain and loss with rs12641823 was not found.

### Hsa-miR-494-3p Targeting rs3749585^CC^ and Downregulating the Expression of Corin

**Figure 3:**
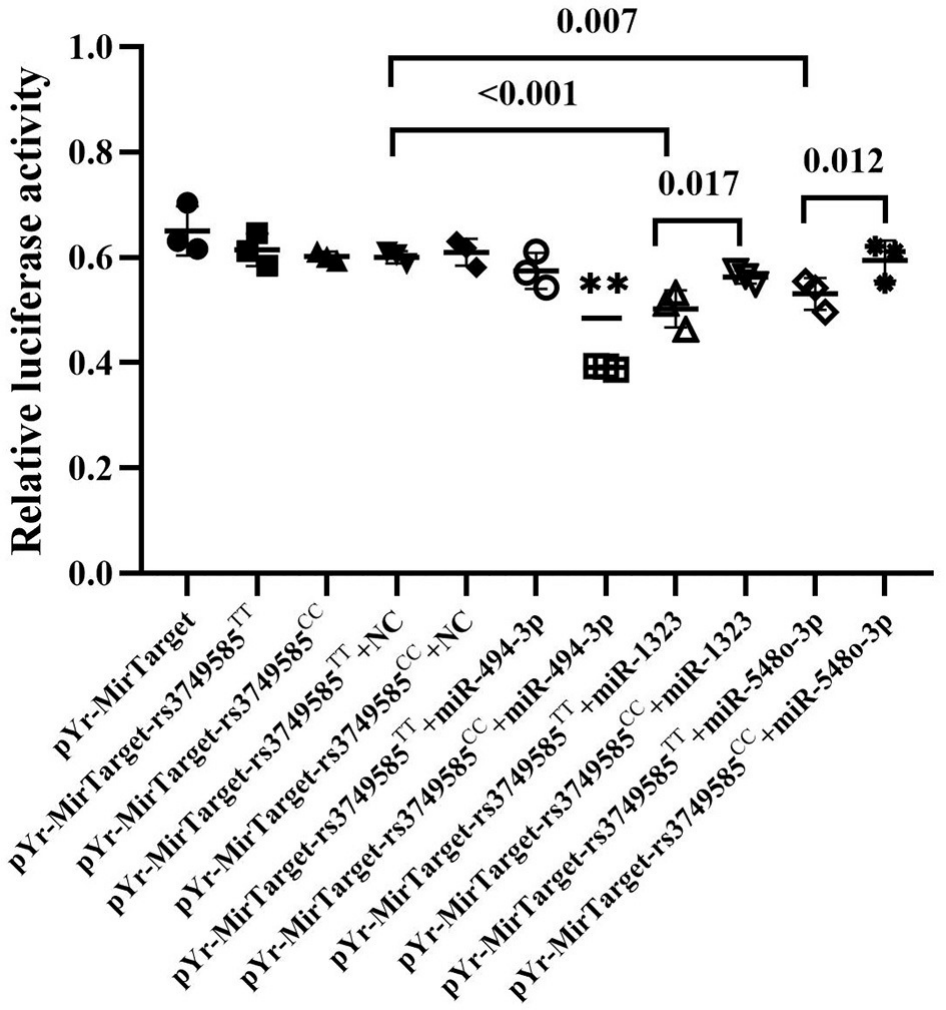
The relative luciferase activity of different miRNAs that gain or lose target with rs3749585^TT/CC^. **A significant decrease in the relative luciferase activity of rs3749585^CC^ reporter was detected, compared with rs3749585^TT^ reporter in the presence of miR-494-3p (*P* < 0.001). A significant decrease in the relative luciferase activity of rs3749585^TT^ reporter was observed, compared with rs3749585^CC^ reporter in the presence of miR-1323 or miR-548o-3p (*P* = 0.017 and 0.012, respectively).

We investigated whether miR-494-3p, miR-548o-3p, and miR-1323 directly target the rs3749585 of *CORIN* using a dual luciferase assay. The luciferase assay revealed that miR-494-3p mimics significantly decreased the luciferase activity of rs3749585^CC^-transfected cells, in comparison to cells transfected with rs3749585^TT^ (*p* < 0.001, Figure 3). A significant decrease in the relative luciferase activity of rs3749585^TT^ reporter, compared with rs3749585^CC^ reporter, was observed in the presence of miR-1323 or miR-548o-3p (*p* = 0.017 and 0.012, respectively, Figure 3). The results demonstrated that miR-494-3p could target gain rs3749585^CC^ and reduce the expression of *CORIN*. MiR-1323 and miR-548o-3p could lose target with rs3749585^CC^ and had no effect on the expression of *CORIN*.

## Discussion

In the present study, we investigated the association between three SNPs in the 3' UTR of *CORIN* and CVDs (EH, AF, HF, and CAD) in a Chinese Han population. We also examined the relationship between the SNPs of *CORIN* and lipid levels. To the best of our knowledge, this is the first time that genetic association between SNPs in the 3'UTR of *CORIN* and CVDs or lipid levels has been observed. Our study demonstrated that rs3749585^T^ and rs4695253^T^ were associated with EH and that rs4695253^T^ was associated with blood levels of HDL and TC. In addition, rs3749585^T^ was significantly associated with protection from AF. We revealed that hsa-miR-494-3p could target rs3749585^CC^ and downregulate the expression of *CORIN* using dual-luciferase reporter assay.

Bioinformatic analysis showed that the three SNPs, rs3749585^T^, rs4695253^T^, and rs12641823^A^, were functional SNPs, and rs3749585^T^ was also a tagSNP. In our study, rs3749585^T^ was significantly associated with the risk of EH after adjusting for covariates sex and age in allelic [*p*_*adj*_ = 0.049; *OR*: 1.113 (95% CI, 1.00–1.238); Table 2] and dominant [*p*_*adj*_ = 0.015, *OR*: 1.233 (95% CI, 1.042–1.459); Table 3] models. A case–control study including 402 patients with hypertension and 406 participants with normal blood pressure was conducted in Liaoning province of China. It found that rs3749585 correlated significantly with hypertension susceptibility only in the dominant model [*p* = 0.019, *OR*: 1.533 (95% CI, 1.073–2.818)] (32), which is consistent with our results. It should be noted that the populations of that study came from the Northeast of China, which is the same geographic region from which the population in our study came. However, another study reported that rs3749585 was found to be unrelated with hypertension in 731 hypertensive patients and 731 controls from Jiangsu Province, located in the south of China [*p* = 0.852, *OR*: 0.98 (95% CI, 0.85, 1.14)] (33). Differing genetic backgrounds may underlie the discrepancy.

Studies have confirmed the abnormal involvement of *CORIN* in hypertension and ventricular hypertrophy. A genotype–phenotype genetic association study demonstrated that *CORIN* T555I/Q568P allele is common in blacks and is associated with higher blood pressure and an increased risk for prevalent hypertension (10). A further study verified that the *CORIN* T555I/Q568P allele can be an independent predictor of left ventricular mass in subjects with elevated systolic blood pressure in self-identified blacks (11). A functional study demonstrated that T555I/Q568P mutant lacking frizzled-like domain 2 had 30 ± 5% activity compared to that of wild type, and the T555I/Q568P variant had a reduced pro-ANP and pro-BNP processing activity compared to that of wild-type. The zymogen activation of the corin variant was impaired significantly (12). Our study also examined the correlation between three SNPs in the 3'UTR of *CORIN* and LVH in the hypertensive group. However, no association was found though rs3749585^T^ and rs4695253^T^ are related to the risk of EH in different inheritance models (Supplementary Tables 3, 4). It is of note that the SNPs rs3749585^T^ and rs4695253^T^ are not located in the frizzled-like domain 2.

**Figure 4:**
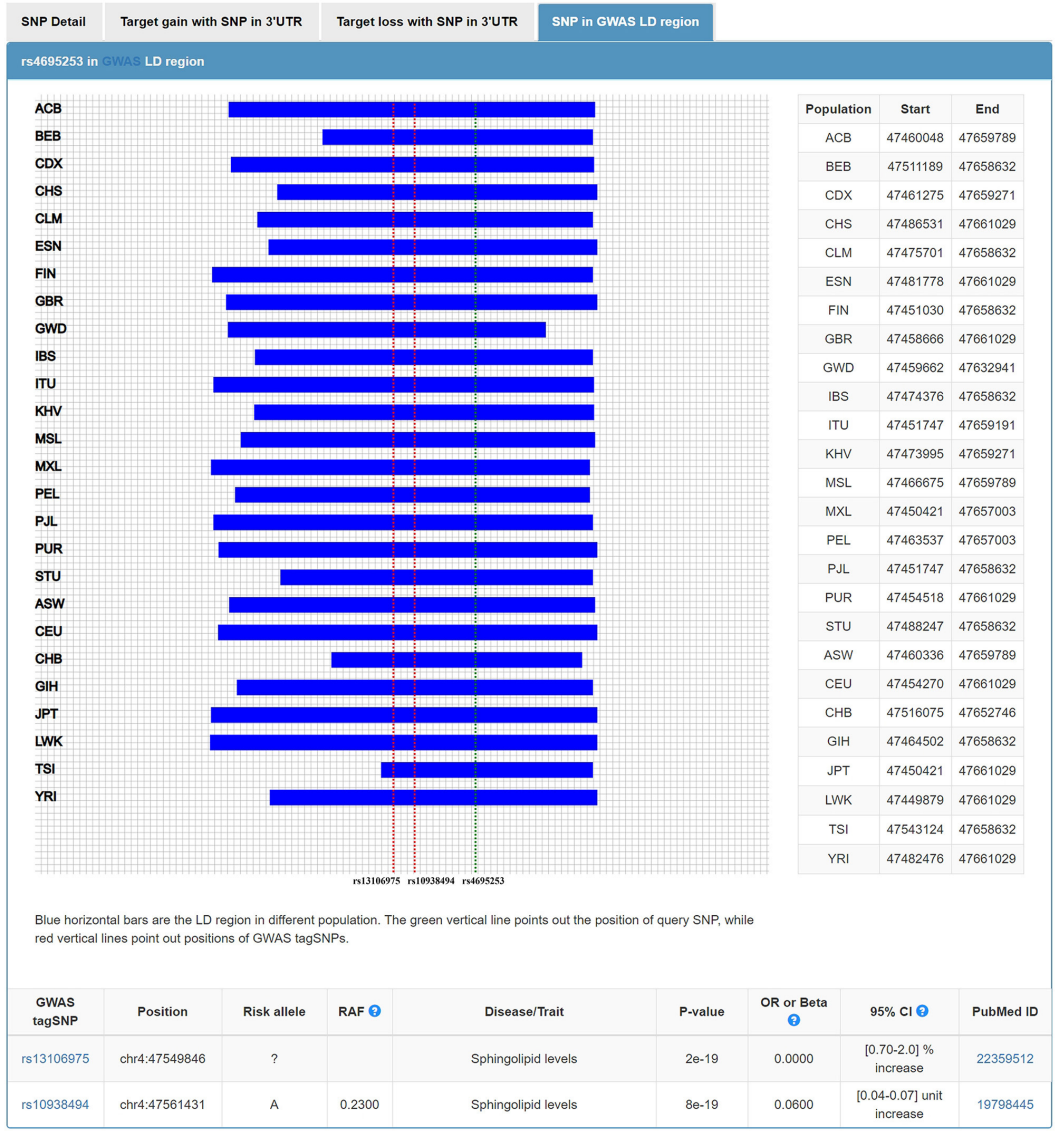
Rs4695253 of *CORIN* and two tag SNPs, rs13106975 and rs10938494 in *ATP10D* on chromosome 4p12, were located in the same linkage disequilibrium region.

In addition to the roles of NPs in water and electrolyte balance, NPs also participate in lipid metabolism (42). The extracellular region of corin consists of two frizzled-like domains, eight LDL receptor (LDLR) repeats, a scavenger receptor-like domain, and a trypsin-like protease domain (43). LDLR repeats 1–4 were important structural elements for corin to recognize its physiological substrate, pro-ANP (44). It is tempting to ask, in addition to this role of LDLR repeats, whether LDLR, as part of the corin structure, participates in the lipid regulation. Wang et al. found that serum soluble corin was significantly and positively associated with dyslipidemia. The findings suggested that serum soluble corin may be a marker or risk factor for dyslipidemia (45). In our study, we investigated the relationship between SNPs of 3'UTR in *CORIN* and the blood levels of lipids. We found that rs4695253^T^ was significantly associated with the levels of HDL and TC in the recessive model after adjusting for covariates sex and age [HDL: *p*_*adj*_ = 0.008, β: 5.053 (95% CI, 1.331–8.775); TC: *p*_*adj*_ = 0.036, β: 15.944 (95% CI, 1.050–30.837); Table 5]. MiRNASNP v3 showed that rs4695253 and the two tag SNPs, rs13106975 and rs10938494 in *ATP10D* on chromosome 4p12, were located in the same linkage disequilibrium (LD) region (Figure 4). Both rs13106975 and rs10938494 were significantly associated with circulating sphingolipid levels (*p*_*cor*_ = 4.45 × 10^−18^, *p* = 5.13 × 10^−4^, respectively) (46, 47). Therefore, the LD region, including rs4695253, may be associated with lipid metabolism. Future investigations are needed to better understand how corin is involved in lipid metabolism.

A study suggested that miR-494-3p may be associated with AF and AF-related stroke (48). Another study showed that miR-494-3p targets JunD and miR-494-3p/JunD is a novel molecular axis involved in obesity-related metabolic cardiomyopathy (49). In our study, bioinformatic analysis predicted that target gain with rs3749585 was for hsa-miR-494-3p and allele C could bind the seed regions of miR-494-3p to regulate the expression of *CORIN* (Figure 2A). Therefore, dual-luciferase reporter assay was performed to verify that miR-494-3p targets rs3749585. The relative luciferase activity of pYr-MirTarget-rs3749585^CC^+miR-493-3p was reduced compared to the activity of pYr-MirTarget- rs3749585^TT^+miR-493-3p (*p* < 0.001, Figure 3). This is consistent with the analyses by bioinformatic software. Notably, allele T was significantly associated with the risk of EH and with the protection of AF. Zhang et al. showed that circulating soluble corin negatively correlated with EH (18). However, Chen et al. found that plasma corin levels positively correlated with AF (19). The inconsistent results suggested that the distinct pathway of corin could be involved in EH and AF. In this study, we did not examine the expression of miR-494-3p in EH and AF patients and cannot rule out the possibility that the expression of miR-494-3p was differentially modulated in the two diseases leading to the different effects of rs3749585 in EH and AF. Further study on the expression of hsa-miR-494-3p in patients with EH and AF is of great significance to understand the heterogeneity of rs3749585 in EH and AF.

Target loss with rs3749585 was predicted for miR-1323 and miR-548o-3p by bioinformatic analysis. In this study, a decrease in the relative luciferase activity of rs3749585^TT^ reporter, compared with rs3749585^CC^ reporter, was observed in the presence of miR-1323 or miR-548o-3p (*p* = 0.017 and 0.012, respectively, Figure 3). However, the roles of the two miRNAs in EH and AF were unclear.

Target gain with rs4695253 was predicted for miR-561-3p and miR-548at-5p. The latter is associated with Parkinson's disease (50), but the roles of the two miRNAs in CVDs have not been studied. Target loss with rs4695253 was predicted for six miRNAs: MiR-1264 was upregulated in the circulation 3 h after ischemia (51) and critical limb ischemia in T2DM (52), and downregulated in neuroglioma (53). MiR-550a-3p was downregulated in myelodysplastic syndromes (54). MiR-550a-3p also plays its tumor-suppressor role by directly repressing ERK1 and ERK2 protein expression, thereby suppressing the oncogenic ERK/RSK cascade to reduce breast cancer cell viability, survival, migration, invasion, tumorigenesis, and metastasis (55). MiR-3184-3p may help to stabilize miR-423-5p in the pericardial fluid in patients undergoing cardiac surgery (56). MiR-200c-5p inhibition could reduce the effects of SnHG12 downregulation on cell viability and apoptosis, without affecting SnHG12 expression levels in renal cell carcinoma (57). However, the underlying mechanisms by which the six miRNAs participate in the development of CVDs through binding to rs4695253 remain to be elucidated.

Further investigation is required to understand the biological function of the SNPs of the 3'UTR of *CORIN* in CVDs. First, the relationship between SNPs in the 3'UTR of *CORIN* and CVDs in the Chinese Han population is the focus of this study; the results need to be validated in other ethnic cohorts. Second, we only tested the associations between SNPs in the 3'UTR of *CORIN* and CVDs. The SNPs in all the exons and exon–intron boundaries of *CORIN* were not tested in our studies. Third, hsa-miR-494-3p, hsa-miR-1323, and hsa-miR-548o-3p were validated to target rs3749585 and regulate the expression of *CORIN* by dual-luciferase reporter assay. However, in view of the different roles of rs3749585 in EH and AF, the expressions of the three miRNAs need to be probed in EH and AF patients. Fourth, the mechanisms whereby the miRNAs participate in the development of EH and lipid metabolism through targeting rs4695253 need to be further studied.

In conclusion, we found significant associations: rs3749585^T^ and rs4695253^T^ and EH, rs4695253^T^ and the levels of TC and HDL, and rs3749585^T^ and AF. No correlation was found between the three SNPs in the 3'UTR of *CORIN* and CAD, HF, or LVH in hypertensive patients. Our work provides an important understanding of the roles of SNPs in the 3'UTR of *CORIN* in susceptibility to EH and AF in the Chinese Han population. Luciferase assay revealed that the relative luciferase activity of rs3749585^CC^-transfected cells was significantly decreased by miR-494-3p, in comparison to cells transfected with rs3749585^TT^. Further study on the expression of hsa-miR-494-3p in patients with EH and AF is of great significance to understand the heterogeneity of rs3749585 in EH and AF. Hsa-miR-494-3p may serve as a potential therapeutic target for EH and AF patients in the future.

## Data Availability Statement

The datasets presented in this study can be found in online repositories. The names of the repository/repositories and accession number(s) can be found in the article/Supplementary Material.

## Ethics Statement

The studies involving human participants were reviewed and approved by Ethics Committee of The First Affiliated Hospital of Dalian Medical University. The patients/participants provided their written informed consent to participate in this study.

## Author Contributions

YL and FC contributed to the conception of the study and manuscript writing. YiZ contributed to the management of blood samples, DNA extraction, and dual-luciferase reporter assay. XY contributed to SNP genotyping. YaZ contributed to the clinical data collection. YutZ and SZ contributed to data entry and organization. SL and YuaZ contributed to data processing and statistical analysis. WZ and JL contributed to bioinformatics analysis. YY and YX proposed critical revision to the manuscript. All authors contributed to the article and approved the submitted version.

## Conflict of Interest

The authors declare that the research was conducted in the absence of any commercial or financial relationships that could be construed as a potential conflict of interest. The reviewer CX declared a shared affiliation, with no collaboration, with of the authors YiZ, to the handling editor at the time of the review.

## Publisher's Note

All claims expressed in this article are solely those of the authors and do not necessarily represent those of their affiliated organizations, or those of the publisher, the editors and the reviewers. Any product that may be evaluated in this article, or claim that may be made by its manufacturer, is not guaranteed or endorsed by the publisher.
