# Supplementary material for: Single-Nucleotide Polymorphisms in the 3' Untranslated Region of CORIN Associated With Cardiovascular Diseases in a Chinese Han Population: A Case–Control Study
Source: Front Cardiovasc Med. 2021 Aug 2;8:625072. doi: 10.3389/fcvm.2021.625072 (PMC8365884; doi:10.3389/fcvm.2021.625072)
Supplement: Supplementary file 1 [file Data_Sheet_1.docx]

| **Supplementary Table 1 Primers used for genotyping variants in the 3’UTR of *CORIN* by high-resolution melt curve** | | | |
| --- | --- | --- | --- |
| Variants | Nucleotide sequence (5’-3’) | Amplicons(bp) | Annealing temperature(℃) |
| rs3749585 | Forward:atggacagaaatgctcaatcgtgc | 166 | 56.6℃ |
|  | Reverse: tcatggttaggcctggcaaaag |  |  |
| rs4695253 | Forward: ttcatccataccaacaaagtcat | 120 | 51.5℃ |
|  | Reverse: ccaatgagaagaaaccagat |  |  |
| rs12641823 | Forward:atggatgaacgcaagaaaactgaaa | 105 | 51.5℃ |
|  | Reverse: ttgcaaaagagaatttagatgagaga |  |  |
| rs3749584 | Forward: cagctacactaaaagaaaatggcct | 98 | 51.5℃ |
|  | Reverse: gcacgattgagcatttctgtcca |  |  |
| 3’UTR, 3 untranslated region. | | | |

**Supplementary Table 2 Hardy-Weinberg equilibrium in case and control groups**

| Group | rs3749585 | | rs4695253 | | rs12641823 | |
| --- | --- | --- | --- | --- | --- | --- |
|  | Chi-sqare | *P* | Chi-sqare | *P* | Chi-sqare | *P* |
| EH | 2.670266099 | 0.102238928 | 4.396909968 | 0.03600411 | 4.356034986 | 0.036878044 |
| AF | 0.479450193 | 0.488671462 | 2.327722714 | 0.127087679 | 0.130832074 | 0.717570872 |
| HF | 0.859226838 | 0.353955146 | 0.000367985 | 0.984695172 | 0.123973634 | 0.724764095 |
| CAD | 2.650145345 | 0.103540429 | 0.390899368 | 0.531827034 | 1.277404897 | 0.258382109 |
| Control | 6.844735684 | 0.008890285 | 0.960111787 | 0.327158715 | 0.06435915 | 0.799734248 |
| LVH | 1.650714012 | 0.198861778 | 3.073811837 | 0.079562711 | 3.994857124 | 0.045639322 |
| There was no deviation from the Hardy-Weinberg equilibrium for rs3749585, rs4695253 and rs12641823 in either case or control groups (*P*> 0.001); rs3749584 (not shown) deviated significantly. EH, essential hypertension; AF, atrial fibrillation; HF, heart failure; CAD, coronary artery disease; LVH, left ventricular hypertrophy. | | | | | | |

**Supplementary Table 3 Analysis of allelic association of SNPs in 3’ UTR of *CORIN* with LVH in hypertensive group**

| SNP | Sample size  (total n=1495) | Risk allele | Frequency | Without adjustment | | With adjustment | |
| --- | --- | --- | --- | --- | --- | --- | --- |
|  | LVH/non-LVH |  | LVH/non-LVH | *P_obs_* | *OR (95% CI)* | *P_adj_* | *OR (95% CI)* |
| rs3749585 | 590/905 | T | 0.477/0.488 | 0.567 | 0.958 (0.827-1.109) | 0.629 | 0.964 (0.832-1.118) |
| rs4695253 |  | T | 0.144/0.157 | 0.304 | 0.895 (0.725-1.105) | 0.407 | 0.914 (0.740-1.130) |
| rs12641823 |  | A | 0.462/0.456 | 0.765 | 1.023 (0.879-1.191) | 0.813 | 1.019 (0.875-1.186) |
| SNP, single-nucleotide polymorphism; 3’UTR, 3 untranslated region; OR, odds ratio; CI, confidential interval; LVH, left ventricular hypertrophy; *P_adj_*, P value for association after adjusting for covariates sex and age by multiple logistic regression analysis using SPSS version 22.0; *P_obs_*, P value for association before adjusting for covariates age and sex by 2×2 contingence tables using PLINK version 1.07. | | | | | | | |

**Supplementary Table 4 Analysis of genotypic association of SNPs in 3’ UTR of *CORIN* with LVH in hypertensive group under**

**different genetic inheritance models**

| SNP | Model | Without adjustment | | With adjustment | |
| --- | --- | --- | --- | --- | --- |
|  |  | *P_obs_* | *OR (95% CI)* | *P_adj_* | *OR(95% CI)* |
| rs3749585 | Dominant | 0.428 | 0.909 (0.718-1.151) | 0.512 | 0.924 (0.729-1.171) |
|  | Recessive | 0.891 | 0.983 (0.767-1.260) | 0.897 | 0.984 (0.766-1.263) |
|  | Additive | 0.560 | 0.957 (0.824-1.111) | 0.624 | 0.963 (0.829-1.119) |
| rs4695253 | Dominant | 0.213 | 0.859 (0.677-1.091) | 0.289 | 0.878 (0.691-1.116) |
|  | Recessive | 0.833 | 1.070 (0.570-2.010) | 0.751 | 1.108 (0.588-2.087) |
|  | Additive | 0.315 | 0.900 (0.732-1.106) | 0.417 | 0.918 (0.746-1.129) |
| rs12641823 | Dominant | 0.353 | 1.117 (0.885-1.410) | 0.361 | 1.115 (0.883-1.409) |
|  | Recessive | 0.602 | 0.934 (0.721-1.208) | 0.541 | 0.922 (0.712-1.195) |
|  | Additive | 0.770 | 1.022 (0.882-1.285) | 0.818 | 1.018 (0.877-1.181) |
| SNP, single-nucleotide polymorphism; 3’UTR, 3 untranslated region; OR, odds ratio; CI, confidential interval; LVH, left ventricular hypertrophy; *P_adj_*, P value for association after adjusting for covariates sex and age by multiple logistic regression analysis using SPSS version 22.0; *P_obs_*, P value for association before adjusting for covariates age and sex by 2×2 contingence tables using PLINK version 1.07. | | | | | |

**Supplementary Table 5 Analysis of allelic association of SNPs in 3’ UTR of *CORIN* with AF, HF and CAD**

| Disease | SNP | Sample size  (total n=3537) | Risk allele | Frequency | Without adjustment | | With adjustment | |
| --- | --- | --- | --- | --- | --- | --- | --- | --- |
|  |  | case/control |  | (case/control) | *P_obs_* | *OR (95% CI)* | *P_adj_* | *OR (95% CI)* |
| AF | rs3749585 | 618/2919 | T | 0.449/0.470 | 0.196 | 0.921 (0.813-1.044) | 0.050 | 0.875 (0.766-1.000) |
|  | rs4695253 |  | T | 0.150/0.138 | 0.276 | 1.103 (0.924-1.317) | 0.543 | 1.058 (0.882-1.270) |
|  | rs12641823 |  | A | 0.442/0.464 | 0.185 | 0.916 (0.804-1.043) | 0.296 | 0.929 (0.809-1.067) |
| HF | rs3749585 | 464/3073 | T | 0.483/0.464 | 0.306 | 1.076 (0.935-1.239) | 0.153 | 1.132 (0.955-1.343) |
|  | rs4695253 |  | T | 0.166/0.136 | 0.019 | 1.262 (1.039-1.532) | 0.080 | 1.237 (0.975-1.570) |
|  | rs12641823 |  | A | 0.450/0.461 | 0.559 | 0.957 (0.828-1.107) | 0.983 | 1.002 (0.841-1.194) |
| CAD | rs3749585 | 715/2822 | T | 0.461/0.467 | 0.695 | 0.976 (0.866-1.100) | 0.255 | 0.926 (0.812-1.057) |
|  | rs4695253 |  | T | 0.132/0.142 | 0.331 | 0.915 (0.766-1.094) | 0.085 | 0.843 (0.694-1.024) |
|  | rs12641823 |  | A | 0.457/0.460 | 0.848 | 0.988 (0.873-1.118) | 0.821 | 1.016 (0.886-1.165) |
| SNP, single-nucleotide polymorphisms; 3’UTR, 3 untranslated region; OR, odds ratio; CI, confidential interval; AF, atrial fibrillation; HF, heart failure; CAD, coronay artery disease; *P_adj_*, P value for association after adjusting for covariates by multiple logistic regression analysis using SPSS version 22.0; *P_obs_*, P value for association before adjusting for covariates by 2×2 contingence tables using PLINK version 1.07. | | | | | | | | |

**Supplementary Table 6 Analysis of allelic association of SNPs in 3’ UTR of *CORIN* with lipid levels**

| Lipid | SNP | Risk allele | Without adjustment | | With adjustment | |
| --- | --- | --- | --- | --- | --- | --- |
|  |  |  | *P_obs_* | *β(95% CI)* | *P_adj_* | *β(95% CI)* |
| TG | rs3749585 | T | 0.085 | (-6.105) (-13.061-0.851) | 0.084 | (-6.105) (-13.024-0.815) |
|  | rs4695253 | T | 0.977 | 4.456 (-4.484-13.397) | 0.273 | 4.978 (-3.918-13.873) |
|  | rs12641823 | A | 0.767 | 0.983 (-5.530-7.496) | 0.900 | 0.416 (-6.061-6.893) |
| HDL | rs3749585 | T | 0.183 | (-0.507) (-1.253-0.239) | 0.138 | (-0.556) (-1.291-0.179) |
|  | rs4695253 | T | 0.032 | 1.227 (0.106-2.349) | 0.065 | 1.046 (-0.064-2.157) |
|  | rs12641823 | A | 0.993 | 0.004 (-0.799-0.806) | 0.890 | (-0.056) (-0.850-0.738) |
| TC | rs3749585 | T | 0.351 | (-1.570) (-4.866-1.727) | 0.286 | (-1.771) (-5.022-1.480) |
|  | rs4695253 | T | 0.072 | 4.130 (-0.365-8.624) | 0.127 | 3.459 (-0.982-7.901) |
|  | rs12641823 | A | 0.865 | 0.281 (-2.945-3.507) | 0.977 | (-0.047) (-3.229-3.136) |
| LDL | rs3749585 | T | 0.689 | (-14.137) (-83.283-55.008) | 0.690 | (-14.064) (-83.142-55.014) |
|  | rs4695253 | T | 0.662 | (-19.483) (-106.736-67.770) | 0.625 | (-21.758) (-109.036-65.520) |
|  | rs12641823 | A | 0.771 | (-9.619) (-74.454-55.215) | 0.829 | (-7.143) (-71.948-57.662) |
| SNP, single-nucleotide polymorphisms; 3’UTR, 3 untranslated region;CI, confidential interval; TG, triglyceride; HDL, high density lipoprotein; TC, total cholesterol; LDL, low density lipoprotein; *P_adj_*, P value for association after adjusting for covariates by multiple logistic regression analysis using SPSS version 22.0; *P_obs_*, P value for association before adjusting for covariates by linear logistic regression using PLINK version 1.07. | | | | | | |
